# Fatty acid profile and effect of *Plukenetia volubilis* L. (sacha inchi) oil on lipid metabolism in rats fed a high-fat diet

**DOI:** 10.1590/1414-431X2025e14684

**Published:** 2025-10-17

**Authors:** T. Mendoza-Almeida, E.G. Ramírez-Roca, S. Suárez-Cunza

**Affiliations:** 1 Department of Human Medicine, San Cristóbal de Huamanga National University, Ayacucho, Peru; 2 Institute for Biochemistry and Nutrition Research (IIBN) “Alberto Guzmán Barrón”, National University of San Marcos, Lima, Peru

**Keywords:** Sacha inchi oil, Polyunsaturated fatty acids, Lipid profile, Cytokines, Lipid mediators

## Abstract

Sacha inchi oil (SIO) is characterized by its high content of polyunsaturated fatty acids (PUFAs), metabolites with beneficial properties on health. The objective was to evaluate the fatty acid (FA) profile of wild SIO and its effect on biochemical parameters of lipid metabolism under a high-fat diet. Twenty-four albino rats were grouped into groups I, II, III, and IV, which ingested *ad libitum* the following diets: conventional diet without supplementation (CDOS), conventional diet supplemented with SIO (CDWS), hyperlipidic diet without supplementation (HDOS), and hyperlipidic diet supplemented with SIO (HDWS) for 6 weeks. The FA content of SIO was assessed by gas chromatography-mass spectrometry. The lipid profile was analyzed by the enzymatic-spectrophotometric method, and cytokines and lipid mediator levels were measured via enzyme-linked immunosorbent assay (ELISA). Α-linolenic acid (ALA) and linoleic acid (LA) constitute 82% of this oil. Two-way ANOVA showed interaction effects between diet and supplement on interleukin (IL)-10 levels, and SIO-supplemented diet significantly decreased triglycerides (TG), very low-density lipoprotein cholesterol (VLDL-C), and the TG/HDL-C ratio levels. Wild SIO is high in ALA and LA. SIO supplementation reduced TG, VLDL-C, and the TG/HDL-C ratio, modulated IL-10, and slightly improved leptin, resolvin-D1 (RvD1), and IL-6 levels.

## Introduction

Alterations in serum and tissue lipid metabolism are risk factors for various diseases, especially chronic diseases such as cardiovascular diseases (CVDs) and cerebrovascular accidents (CVAs), which are also the diseases with the highest mortality and morbidity worldwide (1). According to the World Stroke Organization (WSO), stroke is the second leading cause of death and the leading cause of disability worldwide (2). It is the fourth-leading cause of death and the leading cause of disability in adults in the United States. Stroke incidence and mortality are also increasing in less developed countries where lifestyles are changing, and the population is being restructured (3).

The guidelines for the pharmacological treatment of dyslipidemia in primary health care focus on the correction of total cholesterol (TC), low-density lipoprotein cholesterol (LDL-C), high-density lipoprotein cholesterol (HDL-C), and triglyceride (TG) levels. Emphasis has also been placed on reducing the risk of stroke and CVDs through a series of lifestyle changes (behavioral therapy, dietary changes, and physical activity). In addition to these actions, in some patients with dyslipidemia, pharmacological therapies (statins, cholesterol absorption inhibitors, fibrates, bile acid sequestrants, niacin, and omega-3 fatty acids) are treatment options (4).

However, standard antihyperlipidemic therapy has a low success rate because optimal reductions in LDL-C levels only reduce the risk of CVDs by 30%, leaving a high remaining risk for the development and progression of CVD (5). Therefore, it is necessary to continue introducing and reinforcing preventive measures on lifestyle changes, including diet modifications, which should focus on the consumption of a diet that prevents lipid metabolism disorders to progress to heart disease (6). Specifically, *Plukenetia volubilis* L. (sacha inchi) is considered an important dietary source because of the oil components in its seeds. The main composition of the oil is polyunsaturated fatty acids (PUFAs), including linoleic acid (AL) (ω-6: 12.4-14.1 g/100 g of seed) and Α-linolenic acid (ALA) (ω-3: 12.8-16.0 g/100 g of seed) (7).

ω-6 and ω-3 are essential dietary fatty acids (FAs), and their functions are related to lipid metabolism at the serum and tissue levels. Various studies have reported that SIO has beneficial effects with regard to CVD risk factors. Obese rats fed 2.5 mL of SIO as an emulsion with different ω-3 contents (0.2 and 0.5 g ω-3/day) for eleven weeks presented decreases in TC, TG, and LDL-C levels and increases in HDL-C levels (8). Similarly, in a group of 30 people who received 45 mL of SIO per day for 42 days, at the end of the treatment, the blood TC, LDL-C, and HDL values and especially the TG level significantly normalized compared with the levels of those lipids prior to SIO supplementation (9).

These effects seem to be influenced by the metabolic status of the individual, as in a randomized crossover clinical trial, the intake of 15 mL of SIO together with a high-fat meal reduced the postprandial levels of TC and the inflammatory marker interleukin (IL)-6) in metabolically healthy men. However, in metabolically unhealthy men, this reduction was not observed, while regardless of metabolic status, HDL-C and TG levels did not differ significantly between groups (10). In addition, it has been reported that ω-3 PUFAs and their metabolites have a specific regulatory effect on genes that participate in lipid metabolism (11), especially in the biosynthesis and clearance of TG (12,13).

Taking into account that the studies described were carried out with cultured SIO, the aim of the present study was to investigate the effects of the oil from the seeds of *Plukenetia volubilis* L. of wild origin with a high PUFA content on serum lipid metabolism in a high-fat diet context, in order to contribute to the improvement of human health and the reduction of lipid-related diseases, using both the cultivated and wild forms.

## Material and Methods

### Collection and preparation of SIO

Seeds of *Plukenetia volubilis* L. “sacha inchi” of wild origin were collected from the Villa Aurora community (coord. UTM 18L 666342: 8548650) in the province of La Mar in Ayacucho, Peru. The seeds were collected through intentional sampling and stored in a suitable place at room temperature (RT). Whole healthy seeds were selected. The seeds were disinfected (0.5% sodium hypochlorite) and the husks were removed, followed by another round of disinfection. The seeds were subsequently dried at RT, ground, and cold-pressed for extraction. The oil obtained was placed in dark glass jars and hermetically sealed. Finally, the oil samples were stored in a dry place away from light at a temperature maintained at approximately 21±2°C.

### Determination of the fatty acid profile

The FA profile of SIO was analyzed by the Natural Products Research Unit of Universidad Peruana Cayetano Heredia (UPCH), in accordance with its strict research protocols, using a gas chromatograph (Agilent Technologies 7890, USA) coupled to a mass spectrometer (Agilent Technologies 5975C). For the analysis, 100 mg of oil was initially dissolved in 10 mL of pentane, to which 100 µL of 11.3% KOH in methanol was added, resulting in two phases. The supernatant was injected into a Zebron 810021zb/7MG-G037-10 column (250°C: 100 × 250 × 0.2 µm). The total running time was 37.5 min. The temperature ramp started at 120°C; this temperature was maintained for 1 min, increased by 10°C per minute to 175°C, where it remained for 10 min, increased by 5°C per minute to 210°C, where it remained for 5 min, and increased by 5°C per minute to 230°C, where it remained for 5 min (14).

### Animal experiments

Adult male albino rats (*Rattus norvegicus*) of the Holtzman strain, with an average weight of 302±17 g, were used; the rats were randomly placed in pairs in metal cages. The animals were acclimated to the laboratory conditions for seven days before the experiment and were provided a conventional diet (2.9 kcal/g of feed) and water, both *ad libitum*. The rats were housed under standard laboratory conditions (12-h light/dark cycle) at a temperature of 21°C±2.

### Diet

The diets were prepared using conventional balanced chow for rodents as the base and were acquired from the Food Research and Social Projection Program of the La Molina National Agrarian University (Peru). The caloric content (metabolizable energy) of the diets was 2.9 kcal/g of food, and the nutritional content was as follows: 17.00% protein, 0.92% lysine, 0.98% methionine-cysteine, 6.00% fat, 0.63% calcium, 0.37% available phosphorus, and 12.00% fiber. The conventional diet was composed of cornmeal, soybean meal, extruded soybean meal, wheat byproducts, palm oil, calcium carbonate, dicalcium phosphate, choline chloride (60%), sodium chloride, synthetic amino acids, premixes of vitamins and minerals, antioxidants, and antifungals.

Four different diets were prepared. The conventional diet without SIO supplementation (CDOS) was acquired from the Food Research and Projection Program (2.9 kcal/g). The conventional diet with SIO supplementation (CDWS) contained 1.05 g of SIO for every 10 g of conventional food (3.8 kcal/g). The hyperlipidemic diet without SIO supplementation (HDOS) contained 2.1 g of lard for every 10 g of conventional food (4.8 kcal/g). The hyperlipidemic diet with SIO supplementation (HDWS) contained 2.1 g of lard and 1.05 g of SIO for every 10 g of conventional food (5.7 kcal/g).

The amount of SIO per 10 g of conventional food (1.05 g) was established through a pilot test, which assessed the acceptance and intake of this diet by rats. In addition, this value was chosen to ensure that the amount of oil did not exceed 30% of the total energy. SIO was included in the diet with the aim of simulating the human form of intake, i.e., small amounts used to season food.

### Treatment of the experimental groups

Four groups of six animals each were formed. All of the rats received treatment for 6 weeks; this treatment period was established on the basis of previously published studies (9,10). The groups were as follows: group I, rats fed CDOS (control); group II, rats fed CDWS; group III, rats fed HDOS (hyperlipidemic group); and group IV, rats fed HDWS. Diets and water were provided *ad libitum*.

### Sample collection

After six weeks, the animals were fasted for a period of 12 to 15 h. All the animals were euthanized by decapitation after being anesthetized with ethyl ether. Blood was obtained and centrifuged at room temperature (20-22°C) at 1000 *g* for 15 min to obtain the serum.

### Food intake and body weight measurement

The daily food intake per cage was measured as the difference in weight of the food provided and the food remaining in the trough after 24 h. This difference, which was divided by two, corresponded to the intake of each animal.

Body weight (g) was recorded weekly starting at the end of the conditioning week.

### Determination of the lipid profile

Commercial Wiener laboratory kits based on a coupled enzymatic method were used to determine the serum levels of CT, TG, and HDL-C. Quantification was performed using a Thermo Scientific Genesys UV-VIS (USA) spectrophotometer at a wavelength of 505 nm. VLDL-C and LDL-C levels were determined using the mathematical calculation described by Friedewald (4). The findings verified that the TG levels in our study were within the established reference range (15). Atherogenic indices were also calculated from the obtained values.

### Measurement of interleukins and lipid mediators

The serum levels of IL-6, IL-10, leptin, and resolvin D1 (RvD1) were measured using enzyme-linked immunosorbent assays (ELISA; MyBioSource commercial kits) and a Diateh DR-200Bc microplate reader (Wuxi Hiwell-Diatek Instruments Co., Ltd., China) strictly following the manufacturers’ protocols.

### Statistical analysis

Data distributions were evaluated with the Shapiro-Wilk test of normality (P>0.05), and the homogeneity of variance was determined using the Levene test. Intake and body weight data were evaluated using analysis of variance (ANOVA). Lipid profile, atherogenic indices, lipid mediator, and interleukin data were analyzed by two-way ANOVA, and Tukey's test was used to analyze the main effects. Data processing was performed using the R package programmer and Minitab 16 Statistics.

Data are reported as measures of central tendency and dispersion (means±SD) and area under the curve (AUC).

### Ethical aspects

Good practices were followed for the treatment of laboratory animals and euthanasia, in accordance with the Guide for the Management and Care of Laboratory Animals of the Ministry of Health - National Institutes of Health (16), the Law No. 30407 on the Protection and Welfare of Animals (17), and the recommendations established for the euthanasia of experimental animals that were prepared by the Directorate General XI of the European Commission (18,19). Additionally, approval was obtained from the Ethics Committee of the Faculty of Medicine of the National University of San Marcos in accordance with the Act of the Ethical Evaluation of Research Studies (study code No. 0185-2024).

## Results

### Fatty acid profile of SIO

The SIO from wild *Plukenetia volubilis* L. had a high PUFA content, with 45.19% (tR=29.14 min) ALA (ω-6) and 36.77% (tR=26.89 min) AL, followed by approximately 10% monounsaturated fatty acids (MUFAs), specifically oleic acid (tR=25.04 min), and approximately 8% saturated fatty acids (SFAs), primarily palmitic acid and stearic acid (tR=19.90 and 23.87 min, respectively) (Table 1). These results indicate that the ratio of unsaturated fatty acids to saturated fatty acids (UFAs/SFAs) was 11.5.

**Table 1:** Fatty acid profile of the seed oil of *Plukenetia volubilis* L. (sacha inchi).

	tR (min)	Relative concentration (%)
Palmitic acid (C16:0)	19.9	4.88±0.01
Stearic acid (C18:0)	23.87	3.45±0.01
Stearic acid (C18:1)	25.04	9.12± 0.02
cis-11-Octadecenoic acid (C18:1)	25.2	0.60±0.01
Linoleic acid (C18:2)	26.89	36.77±0.04
Linolenic acid (C18:3)	29.14	45.19±0.03

Data reported as means±SD. In the analysis, 100 mg of sacha inchi oil was used. tR: retention time.

### Food intake and body weight

**Figure 1:**
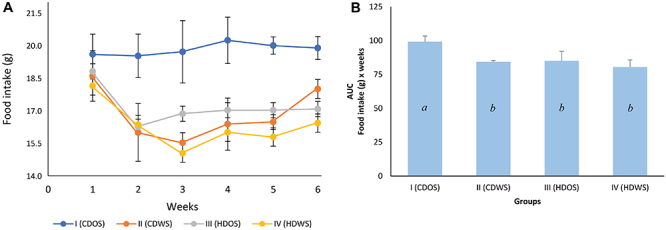
Food intake of rats during the experimental period. **A**, Food intake and **B**, area under the food intake curve (AUC). Group I (CDOS): conventional diet without SIO; group II (CDWS): conventional diet with SIO; group III (HDOS): hyperlipidemic diet without SIO; and group IV (HDWS): hyperlipidemic diet with SIO. SIO: sacha inchi oil. The data are reported as means±SD for n=6 animals per group. P<0.05, different letters indicate statistical significance (one-way ANOVA followed by Tukey's *post hoc* test).

The daily food intake of rats in group I (CDOS), approximately 20 g, was consistent throughout the treatment period. However, in groups II (CDWS), III (HDOS), and IV (HDWS), there was a decrease in intake in the second week; from the third week on, food intake in groups II and IV tended to gradually increase until the end of the study period, while rats in group III maintained their intake from the third week onwards. However, they did not reach the amount ingested by group I (Figure 1A). Specifically, in the AUC of food intake, it was observed that the intake of the groups supplemented with the SIO and the hyperlipidic diet were significantly lower compared to the group I (Figure 1B).

**Figure 2:**
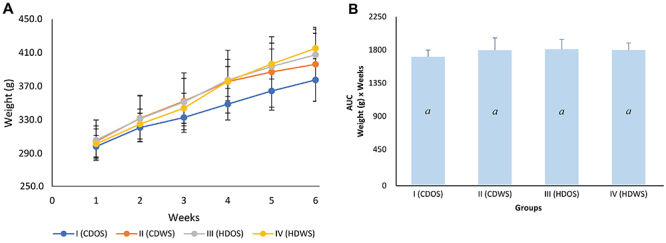
Body weight of rats in the different groups during the experimental period. **A**, Body weight and **B**, area under the body weight curve (AUC). Group I (CDOS): conventional diet without SIO; group II (CDWS): conventional diet with SIO; group III (HDOS): hyperlipidemic diet without SIO; and group IV (HDWS): hyperlipidemic diet with SIO. SIO: sacha inchi oil. Data are reported as means±SD for n=6 animals per group. P<0.05, different letters indicate statistical significance (one-way ANOVA).

Figure 2A shows that the groups fed HDOS (group III), HDWS (group IV), and CDWS (group II) presented a slightly greater increase in weight than did the group fed CDOS (group I) during the 6 weeks of treatment. However, the AUC for the body weight measurements revealed that these increases were not significant (Figure 2B).

### Lipid profile and atherogenic indices

Table 2 presents the results for the different lipid parameters and atherogenic indices. According to the two-way ANOVA results for diet and SIO supplementation, there were no statistically significant interactions among the experimental groups (P>0.05). However, TG levels in the groups fed the control diet were significantly different, with a notable decrease of 57% in TG level when SIO (group II) was added to the diet; a similar result was found for VLDL-C levels.

**Table 2:** Serum lipid profile and atherogenic indices in the different groups of rats.

Lipid parameters	Groups				Interaction	
	I (CDOS)	II (CDWS)	III (HDOS)	IV (HDWS)	Diet × supplement	
					F	P
TC (mg/dL)	79.5±9	75.5±16	92.4±10	94.4±11	0.41	0.529
TG (mg/dL)	82.1±26	35.2±18	53.0±25	40.1±15	3.54	0.075
HDL-C (mg/dL)	45.6±5.6	40.6±4.3	46.5±6.6	51.6±8,3	3.81	0.065
LDL-C (mg/dL)	17.5±10	27.8±13	35.3±11	34.7±8.4	1.55	0.228
VLDL-C (mg/dL)	16.4±5.3	7.04±3.7	10.6±5.2	8.03±3.2	3.54	0.075
TC/HDL	1.77±0.3	1.86±0.4	2.0±0.3	1.87±0.4	0.74	0.400
LDL/HDL	0.39±0.3	0.68±0.3	0.77±0.3	0.70±0.3	2.27	0.148
No HDL-C (mg/dL)	29.0±9.6	34.9±14	49.0±11	43.2±11	0.18	0.679
TG/HDL	1.84±0.7	0.89±0.5	1.2±0.8	0.82±0.4	1.34	0.261

Group I: conventional diet without SIO; group II: conventional diet with SIO; group III: hyperlipidemic diet without SIO supplementation; and group IV: hyperlipidemic diet with SIO supplementation. TC: total cholesterol, TG: triglyceride; HDL-C: high-density lipoprotein cholesterol; LDL-C: low-density lipoprotein cholesterol; VLDL-C: very low-density lipoprotein cholesterol; SIO: sacha inchi oil. Data are reported as means±SD, n=6 per group (ANOVA).

Statistically significant differences were found for the main effects of most of the lipid parameters when considering SIO supplementation and diet. Therefore, Tukey's test was performed (Table 3). The results revealed that the groups that received SIO, regardless of the type of diet, presented significantly lower levels of TG, VLDL-C, and TG/HDL than did the animals that did not receive SIO (P<0.05). In addition, the groups fed a hyperlipidemic diet with or without SIO supplementation presented significantly higher concentrations of TC, HDL-C, and LDL-C than those fed a conventional diet with or without supplementation (P<0.05). However, atherogenic indices such as TC/HDL, LDL/HDL, and non-HDL did not significantly differ (P>0.05).

**Table 3:** Main effects of lipid levels and atherogenic indices.

Lipid parameters	Diet		Supplement	
	CD	HD	OS	WS
TC (mg/dL)	77.52±12.7	93.39±9.6*	85.94±11.6	84.97±16
TG (mg/dL)	58.64±32.8	46.56±21.4	67.53±29.3	37.67±16.5^#^
HDL-C (mg/dL)	43.13±5.4	49.04±7.6*	46.03±5.8	46.14±8.5
LDL-C (mg/dL)	22.67±12.3	34.99±9.4*	26.40±13.8	31.25±10.9
VLDL-C (mg/dL)	11.73±6.6	9.31±4.3	13.51±5.9	7.53±3.3^#^
TC/HDL-C	1.81±0.3	1.94±0.3	1.89±0.3	1.86±0.3
LDL/HDL	0.54±0.3	0.74±0.3	0.59±0.3	0.69±0.3
No HDL-C (mg/dL)	34.39±12.2	44.35±10.4	39.91±11.9	38.83±12.9
TG/HDL	1.37±0.7	1.01±0.6	1.52±0.8	0.86±0.4^#^

CD: conventional diet (n=12); HD: hyperlipidemic diet (n=12); OS: diet without SIO supplementation (n=12); WS: diet with SIO supplementation (n=12). TC: total cholesterol; TG: triglyceride; HDL-C: high-density lipoprotein cholesterol; LDL-C: low-density lipoprotein cholesterol; VLDL-C: very low-density lipoprotein cholesterol. Data are reported as means±SD. *P<0.05 compared with CD and ^#^P<0.05 compared with OS (two-way ANOVA followed by Tukey's *post hoc* test).

### Adipocytokines and lipid mediators

With respect to adipocytokines and lipid mediators (Table 4), a statistically significant interaction effect between diet and SIO supplementation was detected for IL-10 level (F=5.85, P=0.025); for the other three indicators, there were no statistical significance or main effects. Importantly, a tendency toward a decrease in leptin levels was observed between groups III and IV when the hyperlipidemic diet contained SIO. In this same sense, the presence of SIO in groups II and IV showed a slight increase in RvD1 compared to diets without SIO (I and III). Regarding the results for IL-6 levels, which were also not significantly different, group II (CDWS) presented a value between 24 and 37% lower than that observed in the other three groups.

**Table 4:** Levels of adipocytokines and lipid mediators of the different groups.

Lipid mediators and interleukins	Groups				Interaction	
	I (CDOS)	II (CDWS)	III (HDOS)	IV (HDWS)	Diet × supplement	
					F	P
Leptin (ng/mL)	2.70±1.17	3.21±1.63	4.03±2.37	3.24 ±1.48	1.15	0.295
RvD1 (pg/mL)	103.9±30.7	133.0±49.3	85.2±29.3	94.69±20.0	0.5	0.490
IL- 6 (ng/mL)	148.0±61.9	92.8±38.4	122.8±72.8	130.3±54.9	1.73	0.203
IL-10 (ng/mL)	4139±523	3996±682	3062±661	4118±546	5.85	**0.025**

Group I: conventional diet without SIO; group II: conventional diet with SIO; group III: hyperlipidemic diet without SIO; and group IV: hyperlipidemic diet with SIO. SIO: sacha inchi oil; RvD1: resolvin D1; IL-6: interleukin 6; IL-10: interleukin 10. Data are reported as means±SD, n=6 per group. P values in bold indicate significant interactions (P<0.05, two-way ANOVA).

## Discussion

The chromatographic profile of SIO from wild *Plukentia volubilis* L. indicated that the oil contains more than 80% PUFAs (82.0% PUFAs), meeting the NTP 2018 standard (20). SIO from wild *Plukentia volubilis* L. also contains 10% MUFAs and 8% SFAs, resulting in an UFA/SFA ratio of 11.5. In a study by Liu et al. (21), the SIO obtained from cultures of *Plukentia volubilis* L. introduced in China contained 84% PUFAs, 9% MUFAs, and 7% SFAs, resulting in an UFA/SFA ratio of 13.3. Thus, the results of the present study reinforce the nutritional value of the intake of SIO, from both cultivated and wild *Plukentia volubilis* L., owing to its richness in essential PUFAs and its high UFA/SFA ratio. Although its cultivation is currently increasing, there are still areas where this plant has not been cultivated for centuries.

Food intake in the groups supplemented with SIO and those that consumed the hyperlipidemic diet (groups II, III, and IV) was statistically lower compared to group I; however, no significant difference in body weight was observed between the different groups. This decrease in intake could be explained by the increase in fat content in the diets of groups II, III, and IV. Among these fats, the PUFAs present in the SIO are included, which would exert a relatively stronger control over appetite and satiety (22). Among the mechanisms of this control, it has been reported that PUFAs stimulate the release of cholecystokinin (CCK), an enteric hormone related to satiety signals in the brain (23). Furthermore, ALA specifically stimulates the release of another enteric hormone related to satiety and decreased appetite, glucagon-like peptide-1 (GLP-1) (24).

Body weight is also regulated by metabolic products of ALA, such as docosahexaenoic acid (DHA) and eicosapentaenoic acid (EPA), which are considered lipid reducers because they promote beta-oxidation in adipose tissue by inducing AMP-activated kinase (AMPK) expression (25). Additionally, EPA enhances the mitochondrial oxidative activity of uncoupling protein 3 (UCP-3) in adipose and skeletal tissue (26). Furthermore, DHA alone reduces the expression of the genes encoding fatty acid synthase and phosphoenol pyruvate carboxykinase (25). Therefore, SIO, in the course of its metabolism, reduces body fat by inducing the oxidation of fatty acids via a mechanism mediated by thermogenesis.

The groups that were fed diets containing SIO exhibited lower food intake compared to group I, without significantly affecting weight in all groups despite the difference in energetic density.

In the analysis of lipid profiles and atherogenic indices, no interaction between diet and supplement was observed, while in the main effects it was found that the administration of hyperlipidemic diet (HD) with or without supplement, significantly increased the levels of TC, HDL-C, and LDL-C compared to the intake of conventional diet (CD) with or without supplement. This seemed to indicate that the fat content as a source of dietary lipids had a greater effect than the benefit of SIO. In studies on the combined intake of different fatty acids, it has been concluded that both the quality and quantity of fat are relevant in cardiometabolic risk (27). In addition, omega-3 supplementation in diets enriched with SFAs for 6 weeks resulted in a significant increase in TC and LDL-C (28).

The increases in TC and LDL-C are influenced by the synergistic effect of saturated fats and cholesterol present in the HD because these lipids promote the direct hepatic secretion of small LDL-C and the release of its precursors and could even suppress non-LDL-C receptor pathways that are responsible for eliminating smaller LDL-C particles (29).

The elevated HDL-C level indicates that a diet high in saturated fats also generates an increase in this lipoprotein. This is in line with a study reporting that the replacement of carbohydrates for saturated fats in the diet generated more significant increases in HDL-C compared to MUFAs and PUFAs. (30). However, it is necessary to reiterate that the ALA present in the SIO influences the biosynthesis of HDL-C loaded with apoprotein E (apo E), a protein that not only facilitates all the reverse cholesterol transport processes but also promotes the generation of small HDL-C from large ones (α-1 and α-2) (31). Additionally, HDL-C containing APOE attenuates the impact and incidence of cardiovascular diseases (32). Therefore, the composition of this lipoprotein may be a determining factor in its beneficial effects.

Although the LDL/HDL index did not significantly differ between the groups that consumed the CD and those that consumed the HD, an increase of 37% was observed in the animals fed the HD with or without SIO supplementation. This is indicative of the atherogenic effect of the HD.

SIO in lipid metabolism does have a significant role in reducing TG levels and, consequently, VLDL-C and TG/HDL levels compared to groups that were not supplemented with SIO. This decrease demonstrates the regulatory role of SIO at this level, probably due to the high content of omega 3 fatty acids, whose mechanisms include the competitive inhibition of 1,2-diglyceride acyltransferase, which converts diacylglycerides into triacylglycerides, and the activation of receptors by peroxisomal proliferator type α (PPAR-α), which promotes the transcription and translation of enzymes responsible for lipolysis, suppresses the activity of the binding proteins to sterol regulatory element 1 (SREBP-1C), and modulates the expression of genes involved in the *de novo* synthesis of fatty acids and TG (33,34).

The TG and HDL results, expressed as TG/HDL ratio, corroborate the effect of SIO on the serum lipid profile, with a low ratio being notable in the groups fed a diet supplemented with SIO. Thus, the effect of n-3 PUFA supplementation on plasma TGs is the most important among the lipid indicators.

Regarding the lack of significant reduction in TC and LDL-C levels, studies on the effects of PUFAs have reported that these fats only modestly reduce TC and LDL-C levels, and that this is generally evident when these PUFAs replace saturated fats in the diet (12,13). However, as evidenced in the present study, n-3 PUFA supplementation is much more important for plasma TGs. A 2009 study also reported a reduction of approximately 25% in normolipidemic subjects and approximately 50% in hypertriglyceridemic patients (12). A longer period of exposure would probably lead to more pronounced results, although this could also cause unnecessary stress, increased mortality of animals, and consequently the loss of valid samples and data (35). On the other hand, previous investigations carried out over similar or even shorter periods revealed that consumption of SIO decreases hypercholesterolemia, hypertriglyceridemia, and improves the postprandial response to a high fat intake (9,10).

Diets high in lipids can modify the inflammatory state, generally resulting in an increase in proinflammatory cytokines and a decrease in anti-inflammatory cytokines. It is important to highlight that IL-10 level presented a significant interaction between diet and SIO supplementation (F=5.85, P=0.025), showing that the intake of foods with SIO did not alter the level of IL-10 (group II: CDWS and IV: HDWS) but rather regulated it; the results for group I (CDOS) were similar. This result would be influenced by the higher content of ALA (45.19%) than AL (36.77%) in SIO because ALA is classified as anti-inflammatory and pro-resolving, either by direct action or through its oxilipin metabolites. AL promotes the formation of highly potent proinflammatory eicosanoids as a defense mechanism. Therefore, the competition between ALA and AL generates an AL/ALA ratio that determines the amount of ALA converted to EPA, DHA, and anti-inflammatory metabolites (36,37).

HDOS decreased the concentration of IL-10, an effect related to free fatty acids inducing the activation of M1 macrophages, which are responsible for the secretion of tumor necrosis factor alpha (TNF-α), IL-1, and monocyte chemoattractant protein 1 (MCP-1), leading to the inhibition of M2 macrophages and a consequent decrease in IL-10 release. In addition, free fatty acids can act as signal transducer molecules that can bind to Toll-like 2 and Toll-like 4 receptors (TLR 2 and TLR 4). The activation of the latter promotes a decrease in the levels of IL-10 caused by nuclear factor kappa B (NF-kB) (38).

The level of IL-6 in the experimental groups did not significantly differ, although a slight decrease was observed in group II compared with group I, an effect that was also due to n-3 PUFAs, as they suppress the activation of NF-κB, which regulates the generation of inflammatory T cells and inhibits the gene expression of cyclooxygenase 2 (COX-2) (39). Our results are similar to those of a meta-analysis in which supplementation with ALA did not reduce the levels of IL-6 (40). However, another study reported that changes in the level of IL-6 are evident only 1 and 4 h after the administration of a fat-rich diet supplemented with SIO (10).

Although the effect of SIO on the hormone leptin was slightly greater in groups II, III, and IV, the absence of a significant difference between groups can be explained by the absence of significant weight gain. However, with respect to the concentrations of the anti-inflammatory mediator RvD1, the concentrations in group II (with SIO) were slightly greater than those in group I, and greater in group IV (with SIO) than in group III. Thus, a slightly favorable effect was observed for this pro-resolution mediator derived from DHA, which is synthesized from ALA, which is abundant in SIO.

In the present study, it was concluded that SIO from *Plukentia volubilis* L. of wild origin is rich in ALA (ω-3) and AL (ω-6), comparable to that in the cultivated form. The intake of this oil had beneficial effects on the levels of TG, VLDL-C, TG/HDL-C, and IL-10, confirming its mediation effect on lipid metabolism at the serum level, as well as on the inflammatory state, even when it only slightly improves the levels of IL-6, RvD1, and leptin.

### Study limitations

We must recognize some limitations in our study. *Ad libitum* access to the diets allowed varied consumption by each individual, which added variation that could not be controlled owing to the characteristics of each animal, as is the case with humans. However, as this is the first study of SIO from *Plukentia volubilis* L. of wild origin, we decided for free food consumption by the animals. Subsequent studies could consider administering exact doses via the orogastric route. Furthermore, future studies should extend the study time to obtain more information on the effects on serum and tissue biochemical parameters and dyslipidemia, taking into account that such alterations are usually chronic events.

The rat strain used may be questionable. Although most studies are carried out using Wistar and Sprague-Dawley (SD) strains, some studies used the Holtzman strain, which is a derivative of the SD strain. However, the approach involved the consumption of a hyperlipidemic diet, not the strain used.
